# Assessment of the structured clinical interview (SCID) for DSM-5 for somatic symptom disorder in general hospital outpatient clinics in China

**DOI:** 10.1186/s12888-021-03126-0

**Published:** 2021-03-10

**Authors:** Yinan Jiang, Jing Wei, Kurt Fritzsche, Anne Christin Toussaint, Tao Li, Jinya Cao, Lan Zhang, Yaoyin Zhang, Hua Chen, Heng Wu, Xiquan Ma, Wentian Li, Jie Ren, Wei Lu, Rainer Leonhart

**Affiliations:** 1 Department of Psychological Medicine, Peking Union Medical College Hospital, Chinese Academy of Medical Sciences & Peking Union Medical College, Beijing, China; 2 Department of Psychosomatic Medicine and Psychotherapy, Medical Center - University of Freiburg, Faculty of Medicine, Freiburg im Breisgau, Germany; 3 Department of Psychosomatic Medicine and Psychotherapy, University Medical Center Hamburg-Eppendorf, Hamburg, Germany; 4 Mental Health Centre, West China Hospital, Sichuan University, Chengdu, China; 5 Department of Psychosomatic Medicine, Sichuan Provincial People’s Hospital, University of Electronic Science and Technology of China, Chengdu, China; 6 Department of Psychological Medicine, Zhong Shan Hospital, Fudan University, Shanghai, China; 7 Department of Psychosomatic Medicine, Tongji Hospital, School of Medicine, Tongji University, Shanghai, China; 8 Department of Psychosomatic Medicine, Dongfang Hospital, School of Medicine, Tongji University, Shanghai, China; 9 Department of Clinic Psychology, Wuhan Mental Health Center, Wuhan, China; 10 Department of Rehabilitation, General Hospital of Jincheng Anthracite Coal Mining Group Co. Ltd, Jincheng, China; 11 Department of Psychosomatic Medicine, Beijing Hospital of Traditional Chinese Medicine, Capital University, Beijing, China; 12 Institute of Psychology, University of Freiburg, Freiburg im Breisgau, Germany

**Keywords:** DSM-5, Somatization, Psychological tests, Validation

## Abstract

**Background:**

It is still unknown whether the “Somatic symptom disorders (SSD) and related disorders” module of the Structured Clinical Interview for DSM-5, research version (SCID-5-RV), is valid in China. This study aimed to assess the SCID-5-RV for SSD in general hospital outpatient clinics in China.

**Methods:**

This multicentre cross-sectional study was conducted in the outpatient clinics of nine tertiary hospitals in Beijing, Jincheng, Shanghai, Wuhan, and Chengdu between May 2016 and March 2017. The “SSD and related disorders” module of the SCID-5-RV was translated, reversed-translated, revised, and used by trained clinical researchers to make a diagnosis of SSD. Several standardized questionnaires measuring somatic symptom severity, emotional distress, and quality of life were compared with the SCID-5-RV.

**Results:**

A total of 699 patients were recruited, and 236 were diagnosed with SSD. Of these patients, 46 had mild SSD, 78 had moderate SSD, 100 had severe SSD, and 12 were excluded due to incomplete data. The SCID-5-RV for SSD was highly correlated with somatic symptom severity, emotional distress, and quality of life (all *P* < 0.001) and could distinguish nonsevere forms of SSD from severe ones.

**Conclusions:**

This study suggests that SCID-5-RV for SSD can distinguish SSD from non-SSD patients and severe cases from nonsevere cases. It has good discriminative validity and reflects the DSM-5 diagnostic approach that emphasizes excessive emotional, thinking, and behavioural responses related to symptoms.

## Background

Somatic symptom disorder (SSD), formerly known as somatoform disorder (SFD) [1], is one of the most common reasons for visiting physicians [2]. SSD includes somatic symptoms that are not associated with other mental disorders or cannot be medically explained in relation to a patient’s general medical condition [2]. SSD has tremendous relevance for health care systems. Therefore, somatoform symptoms must be reliably classified to improve detection, adequate treatment, and relevant research efforts. In this study, the practical application of structured clinical interviews in this field will be explored.

SFD was introduced in the Diagnostic and Statistical Manual of Mental Disorders-Third Edition (DSM-III) [3] and modified for the DSM-IV [1], but researchers generally agreed that the definition of SFD needed substantial revision [4]. The current diagnostic DSM-5 criteria for SSD encompass the former diagnoses of SFD, pain disorder, undifferentiated somatoform disorder, and, in part, hypochondriasis. Compared with DSM-IV, DSM-5 introduced two major changes. First, the somatic symptoms criterion is no longer limited to medically unexplained symptoms; this eliminates the difficult and subjective distinction between medically explained and unexplained symptoms and mind-body dualism [5, 6]. Second, criterion B now includes positive psychological diagnostic criteria; this criterion now includes excessive thoughts, feelings, and behaviours that are related to the somatic symptoms experienced by the patient or associated with health concerns. For criterion B, the patients had to present at least one of the following: 1) disproportionate and persistent thoughts about symptoms, 2) persistently high anxiety about health or symptoms, and 3) excessive time and energy devoted to the symptoms. Third, according to criterion C, the symptoms have to persist for at least 6 months. Patients with SSD are classified as mild SSD (at least one B criterion), moderate SSD (two or more B criteria), and severe (two or more B criteria plus criterion C).

The DSM system is a common nomenclature to describe psychopathology, but it has been criticized repeatedly [7, 8]. The major critique is that the DSM conceptualizes disorders as categorical entities for which the individuals are dichotomized as being with or without the disorder, which leads to a risk of overmedicalization [7, 8]. Indeed, multiple studies have shown that most psychopathologies are not categorical entities but are rather continuous or dimensional hybrids of dimensional and categorical constructs [9–12].

The structured clinical interview for DSM-5, research version (SCID-5-RV) is a guide for semi-structured interviews for the major DSM-5 diagnoses; the latest version was published by the American Psychiatric Association in 2015 in English [13]. It is still unknown whether the categorical and symptom severity dimensional constructs of the “Somatic symptom disorders and related disorders” module of SCID-5-RV are valid.

Therefore, the aim of this study was to assess the SCID-5-RV for SSD in general hospital outpatient clinics in China. This should provide some basis for the further validation of the SCID-5-RV approach and for its official translation in Chinese.

## Methods

### Study design and settings

This study is a secondary analysis of a dataset that was collected for a previous multicentre cross-sectional study that was conducted from May 2016 to March 2017 in outpatient clinics of nine tertiary hospitals in North, North-Central, East, Central, and West China (Beijing, Jincheng, Shanghai, Wuhan, and Chengdu). The nine participating centres were authoritative centres in the field of psychiatry in China. The modern biomedical setting was represented by the neurology and gastroenterology departments, the Traditional Chinese Medicine (TCM) department represented the TCM setting, and the psychological medicine department represented the psychosomatic medical settings.

### Subjects

A total of 220 patients were recruited from each of the three medical settings. The screening days at each centre were randomly determined. All patients who were admitted to one of the study departments were approached for participation in the study using an informational hand-out. An informed consent form was used to explain the aims of the study to the patients. The patients were fully informed that their participation was voluntary, that the data would be analysed anonymously and that there were no disadvantages in case of refusal to participate.

The inclusion criteria were as follows: 1) > 18 years old; 2) visiting for treatment (i.e., not only picking up a prescription); 3) able to read and write; and 4) signed the written consent form. The exclusion criteria were as follows: 1) language barrier; 2) limited writing skills; 3) cognitive impairment; 4) psychosis; or 5) acute suicidal ideation.

### Assessments

All patients who provided written informed consent filled in questionnaires for general information and quantitative assessment of psychopathology.

In general information acquisition, smoking status was classified as never smoking, former smoker, and current smoker. Drinking status was classified as never drinking, drinking only socially, history of drinking but now abstinent, and currently drinking > 3 days/week. Exercise was classified as > 2 h/day, 1–2 h/day, < 1 h/day, and no physical exercise. The self-evaluation of treatment satisfaction and efficacy was divided into six grades (0–5 points): 0 means “not satisfied with the treatment in the past 6 months at all” or “considered that the treatment in the past 6 months was completely failed”, and 5 means “very satisfied with the treatment in the past 6 months” or “considered that the treatment in the past 6 months was very successful”.

The quantitative assessment of psychopathology included the following: 1) the Patient Health Questionnaire 15 (PHQ-15) was used to assess the number and severity of somatic symptoms; 2) the Somatic Symptom Scale-8 (SSS-8) was used to assess the somatic symptom burden; 3) the Patient Health Questionnaire 9 (PHQ-9) was used to assess depression; 4) the General Anxiety Disorder-7 (GAD-7) was used to assess anxiety; 5) the Whiteley-7 was used to assess health-related anxiety (WI-7); 6) the WHO Disability Assessment Schedule (WHO-DAS 2.0); 7) the Somatic Symptom Disorder B-criteria (SSD-12); 8) the 12-item short-form health survey (SF-12) was used to assess quality of life; 9) questions on health care utilization; and 10) sociodemographic data (age, sex, level of education, marital status, etc.).

The PHQ-15 was validated in Chinese for somatic symptoms [14, 15]. The SSS-8 is a self-rating scale used to quantify the somatic symptom burden of patients in the past week and has been validated in Chinese patients [16]. The severity of depression and generalized anxiety were assessed using the 9-item depression scale PHQ-9 and the 7-item GAD-7, respectively. The Chinese versions of the PHQ-9 and GAD-7 have been shown to valid and reliable [17–19]. Illness anxiety was evaluated using the 7-item WI-7 Chinese version [20]. Population-level and clinical practice health and disability were assessed using the Chinese version of the WHO-DAS 2.0 [21]. Patients’ perception of their symptom-related thoughts, feelings, and behaviours was assessed using the SSD-12, which was developed based on the DSM-5 criteria [22]. The SF-12 captures information on health-related quality of life (QoL) and has been validated in Chinese [23, 24].

### Diagnostic interview

#### Translation of the SCID-5-RV

Given that a Chinese version of the SCID-5-RV was not available at the time, we developed a research version of a semi-structured clinical interview to assess the diagnostic criteria of SSD. The interview was adapted to the English version of SCID-5, which is considered the gold standard measure for DSM diagnoses. The “Somatic Symptom Disorders and Related Disorders” module of the SCID-5-RV was purchased. Some of the authors of this manuscript (KF, A-M M, AT, TL) formed a translation team. In the process of translation, both the SCID-5 SSD standard and the ITC-Test Adaptation Guidelines (Version 2000) of the International Examination Board [25] are taken into account. The specific translation process is clearly explained in the corresponding literature [26]. A final version was accepted and agreed upon by all Chinese researchers after translations were discussed. The Chinese translation did not obtain approval from the APA; therefore, the present study is a preliminary study that establishes the basis for a future Chinese version.

#### The interview processes

All patients underwent an interview (SCID-5-RV) following the criteria of the DSM-5 for SSD. All the research assistants (psychiatrists and postgraduate medical students in psychiatry) were trained on how to administer the SCID-5-RV. The assistants worked under the direct supervision of attending psychiatrists with > 3 years of experience (i.e., the clinical heads of psychosomatic medicine).

Based on the results of the SCID-5-RV, the patients could be diagnosed with SSD in the presence of criteria A and B [2]. Patients with one B criterion were classified as having mild SSD, those with at least two B criteria were classified as having moderate SSD, and those with at least two B criteria and the C criterion were classified as having SSD.

The results of the SCID-5-RV were compared with the results of the other scales. The combination of the PHQ-15 and SSD-12 has been used to diagnose SSD [27].

### Statistical analysis

All data were stored at the University Medical Centre Freiburg. The same centre was responsible for monitoring the project sites and for data analysis. The study was approved by the ethics committees of all participating centres. The approval number at the University Medical Centre Freiburg was S-K276. The data were collected by research assistants between May 2016 and March 2017.

All statistical analyses were performed using SPSS 22.0 (IBM, Armonk, NY, USA). Categorical variables are described as numbers and percentages and were analysed using the chi-square test. Continuous variables are presented as the means and standard deviations and were analysed using Student’s independent-samples t-test. One-way analysis of variance and the LSD post hoc test were used for the comparison of continuous variables among multiple groups. Spearman’s correlation analysis was used to examine the relationship between the SCID-5-RV and the other scales. Two-tailed *P*-values < 0.05 were considered significant. Alpha inflation might be an issue, and to mitigate it, only P-values < 0.001 were considered high-impact differences or correlations.

## Results

### Enrolment.

During the study period, 1269 patients were contacted, and 699 (55.1%) were enrolled. All 699 completed the questionnaires and clinical interviews. Among those who did not participate in the study, 68 (5.4%) met at least one exclusion criterion, and 502 (39.6%) refused to participate (53.0% reported having no time to participate; 29.5% reported having no interest in the study; 8.4% reported a lack of trust in the interviewers; 6.8% reported not feeling well enough to participate; and 2.4% provided other reasons). Among them, 150 were from Peking Union Medical College Hospital, 50 were from Beijing Hospital of Traditional Chinese Medicine, Capital University, 158 were from West China Hospital, Sichuan University, 53 were from Sichuan Provincial People’s Hospital, 50 were from Tongji Hospital of Tongji University, 50 were from Dongfang Hospital of Tongji University, 55 were from Zhongshan Hospital Affiliated to Fudan University, 52 were from Renmin Hospital of Wuhan University, and 81 were from Jincheng People’s Hospital. Two patients had incomplete data and were excluded, and 697 patients finally underwent diagnostic interviews [26].

### Diagnosis of SSD.

**Fig. 1:**
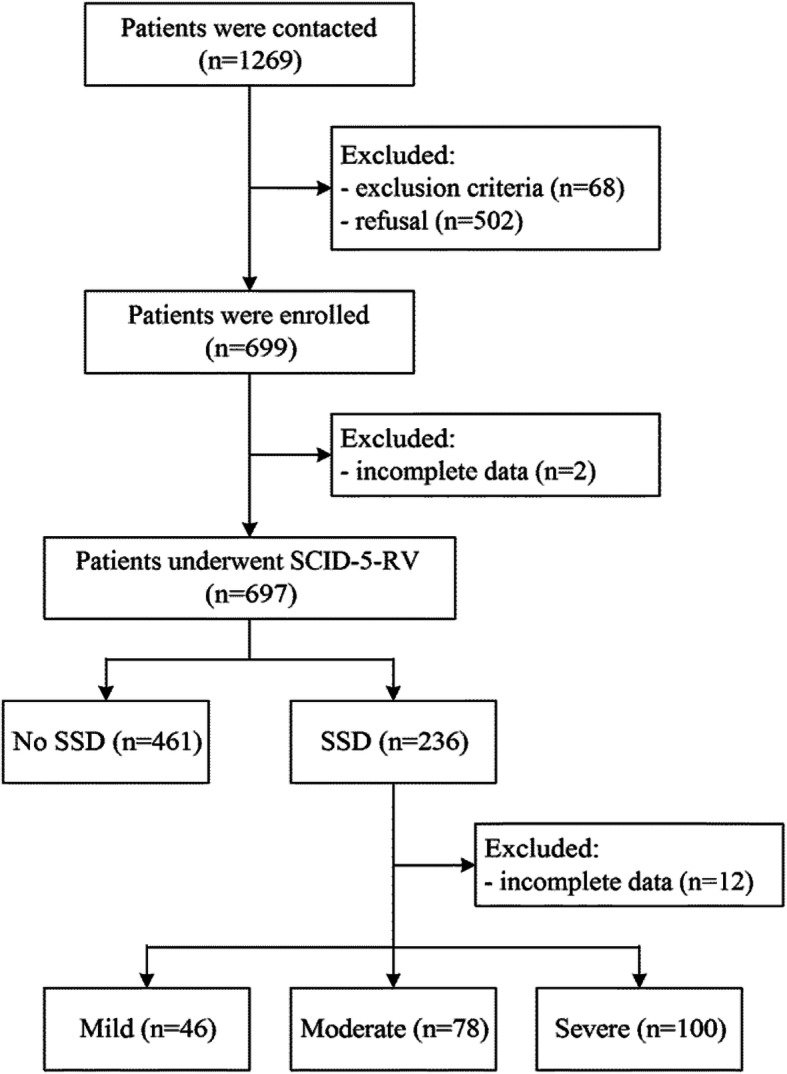
Patient flowchart. SCID-5-RV: Structured clinical interview for DSM-5, research version; SSD: Somatic Symptom Disorder

According to the SCID-5-RV results, 236 (33.8%) were diagnosed with SSD. Of these, 46 (19.5%) had mild SSD, 78 (33.1%) had moderate SSD, 100 (42.4%) had severe SSD, and 12 (5.1%) were excluded due to incomplete data (Fig. 1).

### Sociodemographic characteristics.

When comparing the patients with and without SSD, there were no significant differences between the two groups in demographic variables. The patients with SSD were separated according to severity, and there were no differences among the three groups (all *P* > 0.05) (Table 1).

**Table 1:** Sociodemographic characteristics of the patients

	No SSD (*n* = 473)	SSD (*n* = 224)	P	Mild (*n* = 46)	Moderate (*n* = 78)	Severe (*n* = 100)	P
Age (years)	43.1 ± 14.7	43.0 ± 14.0	0.957	43.5 ± 14.7	40.2 ± 14.2	45.4 ± 13.1	0.052
Sex, female (%)	284(61.6%)	143(60.6%)	0.806	21 (45.7%)	50 (64.1%)	63 (63.0%)	0.088
Health insurance, yes (%)	400(87.7%)	200(85.5%)	0.406	38 (82.6%)	66 (84.6%)	85 (86.7%)	0.800
Residence, urban (%)	386(83.7%)	187(79.6%)	0.174	35(76.1%)	60(76.9%)	84(84.8%)	0.306
Marital status, married (%)	343(74.4%)	163(69.1%)	0.194	31 (67.4%)	49 (62.8%)	77 (77.0%)	0.364
Family income (monthly, Yuans) (%)			0.196				0.850
Low (< 4000)	145(31.7%)	89(38.0%)		17(37.0%)	32(41.6%)	36(36.4%)	
Middle (4000–8000)	162(35.4%)	80(34.2%)		15(32.6%)	24(31.2%)	38(38.4%)	
High (> 8000)	151(33.0%)	65(27.8%)		14(30.4%)	21(27.3%)	25(25.3%)	
Profession, employed (%)	237(51.4%)	105(44.5%)	0.215	25 (54.3%)	34 (43.6%)	39(39.0%)	0.699
Education, university or higher (%)	230(49.9%)	105(44.5%)	0.227	20 (43.5%)	41 (52.6%)	38 (38.0%)	0.239
Exercise in winter, never (%)	123(26.7%)	70(29.7%)	0.670	15 (32.6%)	20 (25.6%)	33 (33.0%)	0.680
Exercise in summer, never (%)	95(20.6%)	54(22.9%)	0.597	9 (19.6%)	14 (17.9%)	29 (29.0%)	0.113
Smoking, never (%)	340(73.8%)	165(70.2%)	0.473	31 (67.4%)	58 (74.4%)	67 (67.7%)	0.165
Alcohol, never (%)	218(47.3%)	127(54%)	0.385	26 (56.5%)	39 (50.0%)	54 (54.5%)	0.908

SSD: somatic symptom disorders

### Clinical characteristics.

When comparing the patients with and without SSD, the patients with SSD had lower treatment satisfaction (2.5 ± 1.8 vs. 3.3 ± 1.6, *P* < 0.001) and worse self-evaluation of the treatment effect (2.4 ± 1.6 vs. 3.1 ± 1.6, P < 0.001). The scores of the questionnaires for symptom severity, emotional distress, and SSD B-criteria in the SSD group were higher than those in the non-SSD group, while the scores for quality of life in the SSD group were significantly lower than those in the non-SSD group (all *P* < 0.05) (Table 2).

**Table 2:** Clinical characteristics of the patients

	No SSD (n = 473)	SSD (n = 224)	P	Mild (n = 46)	Moderate (n = 78)	Severe (n = 100)	P
Satisfaction	3.3 ± 1.6	2.5 ± 1.8	< 0.001	2.7 ± 1.8	2.2 ± 1.7	2.5 ± 1.9	0.344
Effect	3.1 ± 1.6	2.4 ± 1.6	< 0.001	2.6 ± 1.6	2.2 ± 1.5	2.4 ± 1.7	0.366
PHQ-15	8.0 ± 4.8	12.0 ± 5.5	< 0.001	12.0 ± 6.1	10.7 ± 5.0	13.1 ± 5.6	0.019^c^
WHO DAS 2.0	17.3 ± 5.8	22.7 ± 8.5	< 0.001	21.3 ± 8.0	21.1 ± 6.8	24.8 ± 9.8	0.006^b,c^
PHQ-9	6.8 ± 5.8	11.8 ± 6.8	< 0.001	10.4 ± 7.9	11.0 ± 6.0	13.4 ± 6.8	0.012^b,c^
GAD-7	5.1 ± 5.2	9.7 ± 6.1	< 0.001	7.8 ± 6.3	9.0 ± 5.6	11.1 ± 6.3	0.004^b,c^
WI-7	13.2 ± 5.5	21.4 ± 7.4	< 0.001	18.6 ± 7.3	20.7 ± 6.9	23.3 ± 7.2	0.001^b,c^
SSD-12	9.1 ± 9.4	23.6 ± 11.4	< 0.001	17.8 ± 10.8	22.1 ± 10.2	27.5 ± 10.9	< 0.001^a,b,c^
SSS-8	7.0 ± 5.1	12.0 ± 6.5	< 0.001	11.6 ± 7.0	10.8 ± 5.3	13.3 ± 7.0	0.034^c^
SF-12 (PCS)	45.1 ± 8.6	39.1 ± 9.0	< 0.001	41.0 ± 8.2	40.0 ± 9.0	37.4 ± 9.0	0.038^b^
SF-12 (MCS)	44.6 ± 11.5	34.9 ± 11.3	< 0.001	38.6 ± 13.9	34.4 ± 11.6	33.5 ± 9.8	0.044^b^
Number of visits	1.8 ± 0.9	2.2 ± 1.1	< 0.001	1.9 ± 1.0	1.9 ± 1.0	2.6 ± 1.0	< 0.001^a,b,c^

Post hoc analysis was adjusted by LSD. a: P < 0.05 Mild vs. Moderate; b: P < 0.05 Mild vs. Severe; c: P < 0.05 Moderate vs. Severe

PHQ-15: Patient Health Questionnaire 15; WHO-DAS 2.0: WHO Disability Assessment Schedule; PHQ-9: Patient Health Questionnaire 9; GAD-7: General Anxiety Disorder-7; WI-7: Whiteley-7 for health-related anxiety; SSD-12: Somatic Symptom Disorder B-criteria; SSS-8: Somatic Symptom Scale-8; SF-12: 12-item short-form health survey; PCS: physical component score; MCS: mental component score; SCID-5-RV: Structured clinical interview for DSM-5, research version

When comparing the mild, moderate, and severe groups, the SSD-12 scores and numbers of visits to doctors in the last 12 months were significantly different among the three groups. The WHO DAS 2.0, PHQ-9, GAD-7, and WI-7 scores indicated that the mild and moderate groups had significantly lower scores than the severe group, but there was no significant difference between the mild and moderate groups (all *P* < 0.05) (Table 2). The PHQ-15 and SSS-8 scores were significantly lower in the moderate group than in the severe group. The SF-12 PCS and MCS scores indicated that the scores of the mild group were significantly lower than those of the severe group (all P < 0.05) (Table 2).

### Spearman’s correlation analysis.

The results of the SCID-5-RV for SSD diagnosis were highly correlated with somatic symptom severity, emotional distress, and quality of life, which were assessed by the PHQ-15, SSS-8, PHQ-9, GAD-7, WI-7, SSD-12, WHO-DAS 2.0, SF-12 PCS, and SF-12 MCS (all *P* < 0.001). The SCID-5-RV for SSD severity was significantly correlated with scores on the PHQ-9, GAD-7, WI-7, SSD-12, WHO-DAS 2.0, SF-12 PCS, and SF-12 MCS (all P < 0.05) (Table 3).

**Table 3:** Spearman correlation between SCID-5-RV and other assessment scales

	Diagnostic (*n* = 697)		Severity (n = 224)	
	r	P	r	P
PHQ-15	0.355	< 0.001	0.112	0.095
SSS-8	0.385	< 0.001	0.130	0.053
PHQ-9	0.366	< 0.001	0.189	0.005
GAD-7	0.370	< 0.001	0.217	0.001
WI-7	0.530	< 0.001	0.254	< 0.001
SSD-12	0.563	< 0.001	0.339	< 0.001
WHO DAS 2.0	0.351	< 0.001	0.182	0.006
SF-12 (PCS)	−0.310	< 0.001	−0.166	0.013
SF-12 (MCS)	−0.375	< 0.001	− 0.154	0.022

PHQ-15: Patient Health Questionnaire15; SSS-8: Somatic Symptom Scale-8; PHQ-9: Patient Health Questionnaire 9;GAD-7: General Anxiety Disorder-7;WI-7: Whiteley-7 for health-related anxiety; SSD-12: Somatic Symptom Disorder B-criteria; WHO-DAS 2.0: WHO Disability Assessment Schedule; SF-12: the 12-item Short Form Health Survey; PCS: physical composite score; MCS: mental composite score

## Discussion

It is still unknown whether the “SSD and related disorders” module of SCID-5-RV is valid. Therefore, this study aimed to assess the SCID-5-RV for SSD in general hospital outpatient clinics in China. The results suggest that SCID-5-RV for SSD can distinguish SSD from non-SSD patients and severe forms of SSD from nonsevere forms. It has good discriminative validity with other tools and reflects the DSM-5 diagnostic approach that emphasizes excessive emotional, thinking, and behavioural responses related to symptoms.

There were no significant differences in sociodemographic and lifestyle variables between the SSD and non-SSD groups or among the severity groups. These results suggest that sociodemographic factors did not affect the potential diagnostic value of the SCID-5-RV.

In the present study, the SCID-5-RV could identify patients with SSD. A study in China showed that using the SCID-5 led to a frequency of 36.5% for SSD but that there were differences in sociodemographic characteristics among SSD severity groups, with patients with mild SSD having better socioeconomic conditions than those with severe SSD [28]. A Turkish version of the SCID-5-CV showed a κ of 0.65 for SDD [29]. Many questionnaires can be used for the evaluation of SSD [5, 6], but it is considered that the PHQ-15, SSS-8, and SSD-12 provide good diagnostic potential [27]. In the present study, the Spearman correlation of the SCID-5-RV was the strongest with SSD-12, but it was also correlated with PHQ-15 and SSS-8. These results suggest that the Chinese version of the SCID-5-RV could be used for the diagnosis of SSD in Chinese patients. As a diagnostic tool, SCID-5-RV for SSD only has a moderate positive correlation with SSD-12 and WI-7 and a low correlation with other criterion tools such as PHQ-9 and GAD-7. This finding is plausible considering that SCID-5-RV for SSD could distinguish somatic disorders from anxiety and depression disorders well and is more influenced by thoughts, emotions, and behaviours related to symptoms. Similarly, there was a weak correlation between the PHQ-15 and SSS-8 scores. This result may reflect that, under the new diagnostic criteria, the number and load of somatic symptoms have a weaker impact on the diagnostic results.

The PHQ-15, SSS-8, and SSD-12 have also been shown to be able to discriminate among SSD severity groups [27]. Among patients with mild, moderate, and severe SSD, there were significant differences in the SSD-12 scores and the number of doctor visits in the past 12 months. This suggests that with the increase in the severity of SSD, patients have gradually aggravated emotional, thinking, and behavioural problems related to the symptoms and gradually increased use of medical resources. The WHO-DAS 2.0, PHQ-9, GAD-7, and WI-7 scores indicated that the mild and moderate groups were significantly lower than the severe group. This indicates that patients in the severe group have more severe functional impairment, disease belief, anxiety, and depression than those in the nonsevere group. The PHQ-15 and SSS-8 scores indicated that the scores of the moderate group were significantly lower than those of the severe group. This indicates that the somatic symptom load of the severe group was heavier than that of the moderate group. The PCS and MCS scores on the SF-12 indicated that the scores of the mild group were significantly higher than those of the severe group. This indicates that the severe group has more severe functional impairment than the mild group.

The strength of this study was that the outpatient clinics of four departments in nine tertiary hospitals in five Chinese cities participated in this study, resulting in a large sample. Nevertheless, this study had limitations. This study used the convenient sampling method, which may have led to selection bias. This study was cross-sectional and thus lacked follow-up observations of patients to understand the outcome of the disease. The diagnostic value of the SCID-5-RV was not verified. Future studies should formally validate the SCID-5-RV Chinese version.

## Conclusion

In conclusion, this study showed that the SCID-5-RV for SSD could well distinguish SSD patients from non-SSD patients and severe SSD patients from nonsevere SSD patients. There was no significant difference in the clinical characteristics between patients with mild SSD and those with moderate SSD. Both as a diagnostic tool and severity assessment tool, the SSD module of the SCID-5-RV Chinese version had good discriminative validity with other criterion tools, including the PHQ-15, SSS-8, PHQ-9, GAD-7, WI-7, SSD-12, WHO DAS 2.0, and SF-12. Therefore, it is necessary to further explore an evaluation tool suitable for the clinical application of the SSD module of SCID-5-RV in the future.

## Data Availability

The dataset supporting the conclusions of this article is included within the article.
